# Patient trajectories after diagnosis of diffuse large B-cell lymphoma—a multistate modelling approach to estimate the chance of lasting remission

**DOI:** 10.1038/s41416-022-01931-2

**Published:** 2022-08-23

**Authors:** Sara Ekberg, Michael Crowther, Sara Harrysson, Mats Jerkeman, Karin E. Smedby, Sandra Eloranta

**Affiliations:** 1 Department of Medicine Solna, Clinical Epidemiology Division, Karolinska Institutet, Solna, Sweden; 2 Department of Medical Epidemiology and Biostatistics, Karolinska institutet, Solna, Sweden; 3 Red Door Analytics, Stockholm, Sweden; 4 Department of Hematology, Karolinska University Hospital, Solna, Sweden; 5 Division of Oncology, Lund University and Skane University Hospital, Lund, Sweden

**Keywords:** B-cell lymphoma, Epidemiology

## Abstract

**Background:**

Achieving lasting remission for at least 2 years is a good indicator for favourable prognosis long term after Diffuse large B-cell lymphoma (DLBCL). The aim of this study was to provide real-world probabilities, useful in risk communication and clinical decision-making, of the chance for lasting remissions by clinical characteristics.

**Methods:**

DLBCL patients in remission after primary treatment recorded in the Swedish Lymphoma register 2007–2014 (*n* = 2941) were followed for relapse and death using multistate models to study patient trajectories. Flexible parametric models were used to estimate transition rates.

**Results:**

At 2 years, 80.7% (95% CI: 79.0–82.2) of the patients were predicted to remain in remission and 13.2% (95% CI: 11.9–14.6) to have relapsed. The relapse risk peaked at 7 months, and the annual decline of patients in remission stabilised after 2 years. The majority of patients in the second remission transitioned into a new relapse. The probability of a lasting remission was reduced by 20.4% units for patients with IPI 4–5 compared to patients with IPI 0–1, and time in remission was shortened by 3.5 months.

**Conclusion:**

The long-term prognosis was overall favourable with 80% achieving durable first remissions. However, prognosis varied by clinical subgroups and relapsing patients seldom achieved durable second remissions.

## Introduction

Diffuse large B-cell lymphoma (DLBCL) is the most common subtype of malignant lymphoma. It has an aggressive clinical course but also a high chance of treatment response and cure. The addition of rituximab (R) to standard-of-care combination chemotherapy CHOP (doxorubicin, cyclophosphamide, vincristine and prednisolone) has significantly improved patient outcomes since its introduction in the mid 2000s [1–3]. The positive development in DLBCL survival is illustrated by decreasing relapse rates, improved long-term progression-free and overall survival over time [1, 4, 5]. Despite this trend, every fourth DLBCL patient who is treated with curative intent is still expected to experience progressive disease or relapse [6, 7], resulting in a worsened prognosis [8]. On the other hand, achieving a lasting remission for at least 2 years from diagnosis and primary treatment has emerged as a good indicator for a favourable prognosis long term [7, 9, 10].

Population-based cancer survival is often reported using net survival. Although this measure is useful for making comparisons between groups or across time (where mortality due to other causes may differ) they are less useful for understanding real-world probabilities. The aim of this study was to provide real-world summary measures of the chance for lasting remissions which can be useful in risk communication and clinical decision-making. We used multistate models to quantify patient trajectories from remission after first-line treatment for DLBCL, allowing for repeated occurrences of both relapse and remission.

## Materials and methods

### Data

All patients diagnosed with DLBCL between 2007 and 2014 were first identified in the Swedish lymphoma register (SLR) (*N* = 4247 after excluding primary CNS lymphoma, primary mediastinal large B-cell lymphoma and transformed/discordant lymphomas). The SLR contains detailed clinical information on lymphoma characteristics at primary diagnosis, such as stage, WHO performance status, serum lactate dehydrogenase level (S-LDH) and extranodal sites. SLR also contains information on first-line treatment and treatment response. International prognostic index (IPI) was calculated with one point assigned for each of the following factors: age >60 years, Stage III/IV disease, elevated serum-LDH, WHO performance status ≥2 (patient is unable to carry out work and/or bedridden) and one or more extranodal sites [11]. Patients were also classified into risk groups based on the age-adjusted IPI (aaIPI), with one point assigned for each of the factors: Stage III/IV disease, elevated serum-LDH and WHO performance status ≥2.

In 2017–2019, a national review of all medical charts for patients diagnosed with DLCBL 2007–2014 was conducted to confirm recorded treatment responses and relapses and to identify unrecorded relapses. Among relapsed patients, a more detailed data collection on later treatment lines and treatment responses was also performed.

In this study, patients who were treated curatively (i.e., who received three or more cycles of anthracycline-based chemotherapy or equivalent) and responded to the treatment (i.e., who had achieved complete (CR) or partial remission (PR) at their final treatment evaluation with CT or PET-CT) were included (*n* = 2941). Among all patients who experienced a relapse after CR/PR, a more detailed medical record review could be performed in the majority of the cases (471/538, 88%). Reasons for not being included in the detailed data review was either lack of informed consent to review the medical journals for patients who were still alive (*n* = 13, 2%), or that the complete medical records following relapse could not be identified (*n* = 36, 7%).

Information on dates of death was obtained for all 2941 patients by linking the cohort to the national Swedish cause-of-death register through the use of the unique national identity numbers assigned to all Swedish residents [12]. Patients were followed from the end of first-line treatment until death or October 31, 2017 whichever came first. The study has been approved by the regional ethics committee in Stockholm, Sweden.

### Statistical analysis

#### Conceptual modelling framework

Population-based cancer survival is often reported using net survival (e.g., cause-specific survival (CSS) or relative survival (RS)), and summarised at, e.g., 2 or 5 years after diagnosis. Although net survival measures are useful for making comparisons between groups or across time (where mortality due to other causes may differ), the interpretation of net survival is limited to the hypothetical scenario where patients are assumed immune to death from competing causes [13, 14]. An assumption of immortality is also present when estimating, e.g., time to progression or relapse if deaths (due to any cause) are censored in the analysis. To relax this assumption, patient survival estimated in the presence of competing risks can be used as a real-world summary measure of the anticipated prognosis [14, 15].

**Fig. 1:**
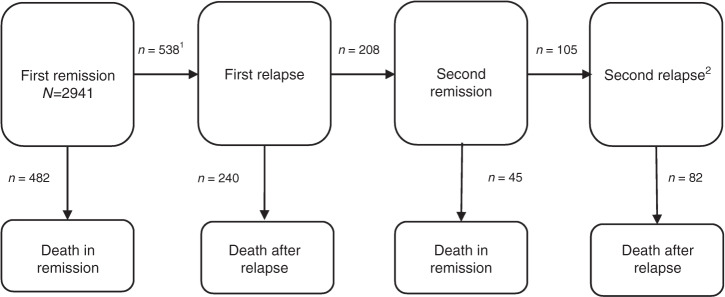
Illustration of the multistate model used to define the transitions (arrow) between different states (boxes). The flow of individuals through the various patient trajectories is indicated by the numbers on each transition. (1) In all, 471 (88%) of 538 relapsing patients had follow-up data beyond the first relapse and could contribute to later transitions. (2) In total, 41 patients responded to later line treatment (following the second relapse) and 14 patients had a third relapse. No patients responded to later line treatment following the third relapse.

Our approach to modelling complex disease pathways and their associated probabilities in the presence of competing risks is via multistate models [16]. For illustration, the multistate model that was used to describe patient trajectories in this study is depicted in Fig. 1. The model was defined by multiple remission and relapse states. The multistate model can be described as a stochastic process with eight states (illustrated by boxes in Fig. 1) and seven possible transitions (illustrated by arrows in Fig. 1).

All patients were included at remission after first-line treatment (starting state). Patients who relapsed transitioned into the “First relapse” state (*n* = 538), from which they could subsequently enter the “Second remission” state if they responded to the relapse treatment and achieved a new remission (*n* = 208). Similarly, patients who experienced a second relapse entered the “second relapse” state (*n* = 105). Patients who progressed on relapse treatment remained in the relapsed state until they eventually died. Each transition between states can be viewed as a survival model accounting for competing events at each transition. The model was used to obtain summary measures for transition probabilities, including probabilities of ever visiting a state, and length of stay at different points of follow-up.

#### Approach to modelling transition rates

The multistate model can be specified as a combination of transition-specific survival models and the possible transitions defined by a transition matrix (Supplementary Fig. 1). In this study, all transition-specific survival models were modelled using flexible parametric models [17], fitted on the log hazard scale using restricted cubic splines to estimate the baseline hazard. The number of degrees of freedom for each transition model was chosen by re-fitting the model with a range of knots (different numbers and localisation) and comparing the Akaike information criterion (AIC) and Bayesian information criterion (BIC) (Supplementary Table 1). Transition probabilities were calculated assuming a Markov renewal or clock-reset process, where time since entry in the current state was used as the underlying time scale for the process. The reason for this approach was that time since e.g. relapse was considered to be of greater importance for the transition probabilities than time since the first remission. Confidence intervals (CI) were calculated using parametric bootstrap [16, 18].

For estimating the probability of remaining in remission by clinical subgroups, we considered eight groups defined by the patients' age at diagnosis (≤60, 61–70, 71–80 and >80 years) combined with their within-group age-adjusted International prognostic index (aaIPI: <2 or ≥2 risk factors). The transition rate to each state was allowed to differ for each clinical subgroup through the inclusion of interaction terms between the group and the baseline hazard function.

We also estimated the impact of sex, Stage (I, II, II, IV), WHO performance status, S-LDH (normal, elevated), number of extranodal sites (0, 1, ≥2) and IPI (0–1, 2–3, 4–5 risk factors) on the difference in the 2-year remission transition probabilities and in the length of stay [16, 19]. For example, with respect to transition probabilities, a difference of −16.1% units comparing patients with Stage IV to patients with Stage I means that the probability of being in remission after 2 years is 16% units lower among Stage IV patients than for Stage I patients. The difference in length of stay gives an estimate of how much the time spent in a particular state is impacted (due to factors included in the model). Continuing the example from above, a difference in the 2-year length of stay of −84.7 days means that patients with Stage IV lose, on average, 84.7 days in the remission state during the first 2 years of follow-up, compared to patients with Stage I. Both types of estimates were adjusted for age and calendar year of diagnosis (both included as continuous variables using restricted cubic splines) and sex.

Lastly, the assumption of proportional hazards was formally tested using likelihood ratio tests by comparing nested models with and without interaction effects between age and year of diagnosis, respectively, and time. For the variables of main interest (sex, stage, WHO performance status, S-LDH, number of extranodal sites and IPI) we included interaction terms with time to accommodate non-proportional hazards irrespective of the level of significance in order not to impose restrictions to the transition rates.

All statistical analyses were performed using Stata v.17 (StataCorp. 2019. Stata Statistical Software: Release 17. College Station, TX: StataCorp LLC) and Stata packages merlin and multistate. More details of the statistical analysis and modelling approach, together with STATA code and a simulated dataset, is available in the Statistical Appendix.

## Results

A total of 2941 patients who responded to first-line curative treatment were included in the study. The median age at diagnosis was 67 years and 44% were women (Table 1). The vast majority received CHOP in combination with rituximab (91%) and of all patients, 90% completed at least six cycles. The median follow-up was 5 years (range: 0–10.6 years). Of the 538 (18%) patients who experienced a relapse during follow-up, 385 (72%) relapsed within 2 years, and 33 (6% of relapses, 1% of all patients) relapsed after more than 5 years. Among the patients who relapsed and had their relapse records reviewed (471), 208 (44%) responded to relapse treatment, i.e., had a second remission, and out of those, 105 (50%) later had a second relapse.

**Table 1:** Clinical characteristics for 2941 patients diagnosed with Diffuse large B-cell lymphoma in Sweden between 2007 and 2014, and who achieved complete/partial remission after first-line curative treatment (at least three cycles).

	Total, *n* (%)	Relapse during follow-up, *n* (%)	Dead during follow-up, *n* (%)
Total	2941	538	887
Age at diagnosis			
Median (range)	67 (18–99)	68.5 (20–93)	74 (23–99)
<50	391 (13)	46 (9)	29 (3)
50–59	393 (13)	75 (14)	61 (7)
60–69	881 (30)	165 (31)	201 (23)
70–79	865 (29)	163 (30)	340 (38)
80+	411 (14)	89 (17)	256 (29)
Sex			
Male	1 656 (56)	321 (60)	511 (58)
Female	1 285 (44)	217 (40)	376 (42)
Ann Arbor stage			
I	651 (22)	58 (11)	149 (17)
II	635 (22)	90 (17)	163 (18)
III	625 (21)	133 (25)	199 (22)
IV	990 (34)	249 (46)	359 (40)
Unknown	40 (1)	8 (1)	17 (2)
Serum lactate dehydrogenase, S-LDH			
Normal	1261 (43)	164 (30)	339 (38)
Elevated	1638 (56)	365 (68)	529 (60)
Unknown	42 (1)	9 (2)	19 (2)
ECOG/WHO performance status			
0—Asymptomatic	1544 (53)	242 (45)	346 (39)
1—Symptomatic but completely ambulatory	978 (33)	188 (35)	361 (41)
2—Symptomatic, <50% in bed during the day	234 (8)	61 (11)	100 (11)
3—Symptomatic, >50% in bed, but not bedbound	133 (4)	33 (6)	53 (6)
4—Bedbound	34 (1)	10 (2)	20 (2)
Unknown	18 (1)	4 (1)	7 (1)
Number of extranodal sites			
0	1590 (54)	248 (46)	436 (49)
1	944 (32)	191 (36)	309 (35)
>1	407 (14)	99 (18)	142 (16)
International prognostic score, IPI			
0	238 (8)	10 (2)	10 (1)
1	724 (25)	98 (18)	175 (20)
2	831 (28)	139 (26)	249 (28)
3	686 (23)	173 (32)	255 (29)
4	311 (11)	83 (15)	131 (15)
5	59 (2)	17 (3)	29 (3)
Unknown	92 (3)	18 (3)	38 (4)
Total	2941	538	887
Age-adjusted International prognostic score, aaIPI			
Age ≤70 aaIPI<2	972 (33)	111 (21)	125 (14)
aaIPI ≥2	771 (26)	191 (36)	197 (22)
Age >70 aaIPI <2	694 (24)	101 (19)	306 (35)
aaIPI ≥2	480 (16)	132 (25)	251 (28)
Missing	24 (1)	3 (1)	8 (1)
Primary treatment			
R-CHOP	2685 (91)	470 (87)	824 (93)
R-CHOEP	176 (6)	49 (9)	34 (4)
R-DA-EPOCH	7 (0)	1 (0)	1 (0)
R-CEOP	34 (1)	7 (1)	17 (2)
R-HD-Mtx containing regimens	30 (1)	8 (1)	7 (1)
Other potentially curative	9 (0)	3 (1)	4 (0)
No. of cycles received			
3–5	297 (10)	40 (7)	160 (18)
6	2455 (83)	458 (85)	674 (76)
>6	189 (7)	40 (7)	53 (6)

**Fig. 2:**
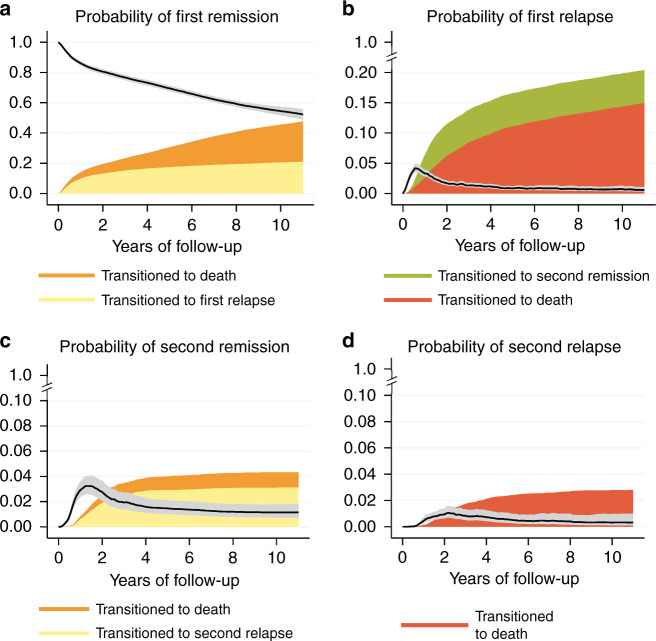
Probabilities of being in a given state by time since first remission (in years) plotted together with the probability of having transitioned to a subsequent state (i.e., where did the patients leave to). Panel **a** shows the probability of being in remission and the probability of subsequently having transitioned into either first relapse or death. Panel **b** shows the probability of being in the state “first relapse” and the probability of subsequently having transitioned into either second remission or death or first relapse. Panel **c** shows the probability of being in “second remission” after relapse treatment and the probability of subsequently having transitioned into a second relapse or death. Panel **d** shows the probability of being in a second relapse and subsequently having transitioned into death (the only possible transition). Note: in order to provide greater detail to the figure, the scale of the y axis differs between panels.

In the entire study cohort, the proportion of patients who remained in the first remission dropped substantially during the first year after treatment completion (from 100% at the start of follow-up to 86.1% (95% CI: 84.6, 87.4) (Fig. 2a). At 2 years, 80.6% (95% CI: 78.9–82.2) of the patients remained in first remission and an additional 2.8% (95% CI 2.1, 3.6) were in the second remission after relapse treatment. A total of 13.2% (95% CI: 11.9–14.6) had relapsed, whereas 6% had died at 2 years whilst in the first remission. From the second year and onwards, the annual decline of patients still alive and in first-line remission, stabilised to ~3–4% units per year (Fig. 2a). Five years after first-line treatment completion, 69.5% (95% CI: 67.7–71.3) of all patients were alive and in a lasting remission from first-line treatment and in total 17.4% (95% CI: 16.0–18.9) had relapsed.

The relapse risk peaked around 7 months after first remission, and at 2 years after remission, 6.2% (95% CI: 5.2–7.5) of the entire cohort had died following a relapse (without achieving a second remission) while 5.3% (95% CI: 4.2–6.6) had achieved a second remission. For patients achieving a second remission, the majority transitioned into a second relapse (Fig. 2c).

**Fig. 3:**
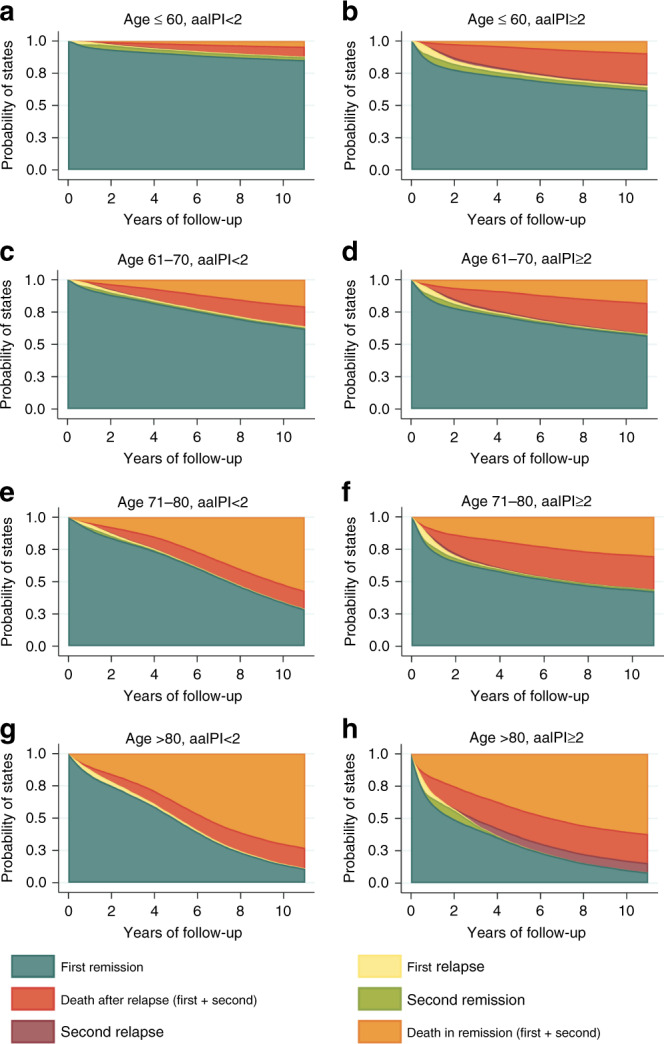
Probabilities of being in a given state at different times since first remission (in years) by age group and age-adjusted international prognostic index (aaIPI). **a** Age ≤60 years, aaIPI <2; **b** age ≤60 years, aaIPI ≥2; **c** age 61–70 years, aaIPI <2; **d** age 61–70 years, aaIPI ≥2; **e** age 71–80 years, aaIPI <2; **f** age 71–80 years, aaIPI ≥2; **g** age >80 years, aaIPI <2, and **h** age >80 years, aaIPI ≥2.

The prognosis varied by clinical risk factors. When stratifying patients by age and age-adjusted IPI score (aaIPI < 2 versus aaIPI ≥2), the initial drop of patients in remission was more pronounced in the latter group (irrespective of age at diagnosis). For example, 88.4% (95% CI: 85.4–90.9) and 84.0% (95% CI: 80.4–87.0) of patients aged 61–70 and 71–80 years with aaIPI<2 remained in remission after two years, compared to 78.3% (95% CI: 74.3–81.2) and 65.7% (95% CI: 60.8–70.3) for patients with the same age and aaIPI≥2 (Table 2). This initial drop in the probability of remission was a reflection of the relapse risk early during follow-up (Fig. 3). At 2 years, a total of 8.6% (95% CI: 5.6–13.0) and 9.2% (95% CI: 6.6–12.5) of patients aged 61–70 and 71–80 years with aaIPI<2 had experienced a relapse compared to 16.7% (95% CI: 13.3–20.7) and 21.2% (95% CI: 17.4–25.5) of patients with aaIPI ≥2. Naturally, the proportion of patients who died when in remission from their DLBCL was higher in the older age groups.

**Table 2:** Two-year probabilities (%) of being in (b) or having visited (v) a given state by age group and age-adjusted IPI.

	2 years after treatment completion	
Age < =60 years	Age-adjusted IPI < 2	Age-adjusted IPI ≥ 2
First CR/PR (b)	93.4 (68.3, 99.0)	77.9 (71.1, 83.3)
Second CR/PR (b)	3.2 (1.1, 5.2)	4.4 (1.6, 7.1)
First relapse (v)	6.2 (2.3, 10.0)	20.7 (13.4, 28.1)
Second relapse (v)	0.8 (0, 1.7)	3.5 (1.6, 5.6)
Dead (first remission)	4.7 (0.0, 9.8)	1.4 (0.3, 6.2)
Dead (second remission)	0.3 (0, 0.8)	1.0 (0.1, 1.9)
Dead (first relapse)	1.0 (0, 2.2)	9.1 (4.7, 13.6)
Dead (second relapse)	0.3 (0, 0.9)	1.3 (0.3, 2.3)
**Age 61–70 years**	**Age-adjusted IPI** < **2**	**Age-adjusted IPI** ≥ **2**
First CR/PR (b)	88.4 (85.4, 90.9)	78.3 (74.3, 81.2)
Second CR/PR (b)	1.6 (0.7, 3.9)	3.2 (1.6, 6.1)
First relapse (v)	8.6 (5.6, 13.0)	16.7 (13.3, 20.7)
Second relapse (v)	1.3 (0.6, 2.9)	3.2 (1.8, 5.6)
Dead (first remission)	3.0 (1.7, 5.2)	5.1 (3.1, 8.0)
Dead (second remission)	0.5 (0.1, 1.4)	1.3 (0.6, 3.0)
Dead (first relapse)	3.4 (1.7, 6.5)	6.9 (04.3, 10.6)
Dead (second relapse)	0.7 (0.3, 1.5)	1.6 (0.8, 3.2)
**Age 71–80 years**	**Age-adjusted IPI** < **2**	**Age-adjusted IPI** ≥ **2**
First CR/PR (b)	84.0 (80.4, 87.0)	65.7 (60.8, 70.3)
Second CR/PR (b)	1.5 (0.6, 3.5)	2.5 (1.0, 5.9)
First relapse (v)	9.2 (6.6. 12.5)	21.2 (17.4, 25.5)
Second relapse (v)	0.8 (0.4, 1.9)	4.9 (2.7, 8.7)
Dead (first remission)	6.8 (4.8, 9.6)	13.1 (9.9, 17.2)
Dead (second remission)	0.9 (0.4, 2.3)	0.4 (0.1, 2.9)
Dead (first relapse)	4.0 (2.2. 7.2)	11.4 (7.9, 16.4)
Dead (second relapse)	0.5 (0.2, 1.2)	3.1 (1.7, 5.6)
**Age** > **80 years**	**Age-adjusted IPI** < **2**	**Age-adjusted IPI** ≥ **2**
First CR/PR (b)	74.9 (68.9, 80.2)	49.4 (41.0, 57.9)
Second CR/PR (b)	0.2 (0, 0.1)	6.8 (0, 16.3)
First relapse (v)	9.3 (6.2, 13.8)	26.3 (19.0, 35.3)
Second relapse (v)	0.3 (0, 2.1)	0.6 (0.0, 7.9)
Dead (first remission)	15.7 (11.6, 21.0)	24.3 (17.6, 32.5)
Dead (second remission)	0.0 (0, 0.8)	0.7 (0, 5.1)
Dead (first relapse)	5.6 (3.1, 9.8)	17.1 (7.4, 35.8)
Dead (second relapse)	0.2 (0, 1.2)	0 (0, 4.8)

**Fig. 4:**
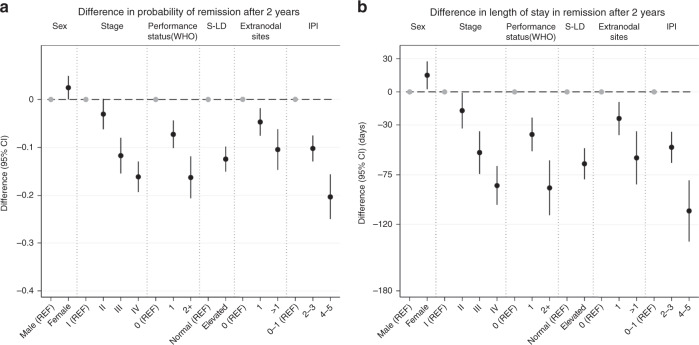
Impact of clinical patient and DLBCL factors on the probability of being in remission and length of stay. The clinical characteristics are contrasted in terms of the difference in the 2 years remission transition probability and length of stay in days. All contrasts are adjusted for age at diagnosis, calendar year of diagnosis and sex at (**a**) difference in transition probability at 2 years (**b**) difference in length of stay at 2 years.

The probability of remaining in first-line remission lasting 2 years or longer was slightly higher for women compared to men (Fig. 4). However, advanced stage (Stage III–IV), performance status ≥1, elevated S-LDH, one or more extranodal sites and IPI score >1 were associated with lower probability of remission at 2 years (after adjustment for age at diagnosis, calendar year and sex). For example, the chance of a lasting remission (at least 2 years) was reduced by 10.3% units for patients with IPI scores of 2–3 (95% CI: −12.9, −7.6)) and by 20.4% units (95% CI: −25.0, −15.7) for those with IPI scores 4–5 compared to those with IPI scores of 0–1.

To translate these probabilities into the amount of time lost in the remission state, we estimated the impact of the clinical factors on the 2-year difference in length of stay. As an example, across the first 2 years of follow-up, poor performance status (≥2) and Stage IV disease both shortened the time in remission by ~3 months compared to asymptomatic patients and Stage I patients, respectively (Fig. 4 and Supplementary Table 1). The patients with the highest IPI scores [4, 5] lost 3.5 months (out of a potential 2 years) in remission (difference in length of stay: −107 days 95% CI: −135.2, −80.0), and patients with IPI scores 2–3 lost 1.6 months (difference in length of stay: −50.1 days, 95% CI: −64.1, −36.1) compared to patients with the lowest IPI score.

## Discussion

Overall, DLBCL patients achieving remission following first-line immunochemotherapy have a good long-term prognosis. We found that 8 of 10 patients were alive and relapse-free at 2 years after end of treatment. However, 13.2% had experienced a relapse, leading to a much worse prognosis. In fact, only 40% of those who relapsed within 2 years also achieved a second remission in that time (5.3% of the whole cohort), and the majority of patients achieving a second remission later transitioned into a new relapse. We also quantified the impact of well-known risk factors on the real-world probability of remaining in remission at 2 years after treatment and found that patients with, e.g., the highest IPI score [4, 5] had a reduced probability by 20% units and also stayed in remission for 3.5 months shorter than patients with the lowest IPI scores (0–1).

The estimated relapse risks from the multistate model were well in line with previously reported numbers in patients achieving first-line remission [1, 10]. The majority of relapses in DLBCL occur within the first 2 years [10, 20, 21] and several studies have pointed to the importance of reaching 2 years of relapse-free disease as a milestone for a future favourable prognosis. In fact, several population-based studies have shown that patients reaching this important milestone have a life expectancy that resembles that of the general population, with only minimal life loss thereafter [7, 9, 10]. These findings have led to changes in several international clinical guidelines of recommended follow-up schemes where the previous recommendation of 5 years of clinical follow-up has been shortened to 2 years for patients remaining in remission at that point [22–24].

The difficulties in treating relapsing patients, and the poor prognosis after relapse, are reflected in this study by the relatively low proportion of patients who enter a second remission as well as the fact that the majority of patients in the second remission transitioned into a second relapse. The standard-of-care for younger and fit patients is still salvaged multiagent chemotherapy followed by consolidation with autologous stem cell transplantation (ASCT) at first relapse. However, about half of the patients are not eligible for such aggressive treatment [25] and only half of those who are eligible eventually undergo ASCT [26]. Of those who complete the treatment 35–45% relapse again [27–29]. The prognosis for patients that are ineligible for ASCT is poor [30]. More recently, the standard-of-care for patients with early relapse or primary refractory disease has been challenged, as CAR-T (chimeric antigen receptor T cell) therapies have shown benefits compared to ASCT (ZUMA-7, Locke F NEJM 2021). CAR-T cell therapy has been available for selected in Sweden at second or later relapses in the last few years, but was not in use in routine care during the study period. Therefore, the current population-based results serve as benchmark of outcomes before the CAR-T era.

The probability of remaining in remission, obtained via this multistate modelling framework, is closely related to measures widely used in clinical trials: progression-free survival (PFS) (defined as time from randomisation to first progression or death from any cause) and disease-free survival (DFS) (defined as time from randomisation to disease recurrence or death from any cause) [31]. However, the multistate model goes further, as it is not limited to the estimated time to first event. In contrast to studies that have investigated patient outcomes in isolation (e.g., relapse or death), we adopted a multistate model approach to gain insights into the interplay of events that the patients may encounter, including death, second remission and second relapses.

Even in this large population-based cohort, the numbers of patients in remission after the first relapse and subsequently experiencing a second and third relapse were small, which prevented us from exploring in more detail the role of prognostic factors beyond the second relapse in this multistate model approach. Another limitation was that the Swedish lymphoma register does not record molecular data, e.g. cell of origin, or MYC/BCL-2/BCL-6 translocations. Even so, the IPI score can be viewed as a surrogate for biological heterogeneity [25, 32] and although it was developed in the 1990s [11] it is still used in clinical practice and has shown to be a robust clinical prognostic score also in the rituximab era [33].

By using registered data in combination with new methods for analysing the course of disease events we can gain understanding and provide measures that are useful for risk communication and health care planning. In this population-based study, we present a comprehensive overview of the real-world prognosis and patient trajectories for patients in remission after DLCBL in the rituximab era. Our results illustrate how the probability of a lasting remission vis-a-vis the risk for relapse and death evolves as a function of follow-up time and by established prognostic factors.

To conclude, we found that the prognosis for patients responding to first-line treatment was overall favourable, as over 80% had durable first remissions of at least 2 years. However, more than one in eight patients are expected to relapse before reaching this milestone and a majority of those reaching a second remission later transition into a second relapse.

## Supplementary information

Stata code

Statistical appendix

Simulated data

Reproducibility checklist

## Data Availability

Research data are not shared due to secrecy agreements made with the authorities that maintain the original data sources.
