# Supplementary material for: Patient trajectories after diagnosis of diffuse large B-cell lymphoma—a multistate modelling approach to estimate the chance of lasting remission
Source: Br J Cancer. 2022 Aug 23;127(9):1642–9. doi: 10.1038/s41416-022-01931-2 (PMC9596493; doi:10.1038/s41416-022-01931-2)
Supplement: Supplementary file 2 — Statistical appendix [file 41416_2022_1931_MOESM2_ESM.pdf]

Statistical appendix

This appendix describes the multistate model (figure 1) and the analysis carried out to estimate transition probabilities and length of stay in the different disease states among Swedish patients diagnosed with DLBCL 2007-2014 and in remission after first line treatment. To further illustrate the flexibility and usefulness of the multistate model we provide example STATA code together with a simulated data set.

The multistate model

The multistate models can be described as stochastic processes: the model $\{Y_a(t), t \geq 0\}$ has eight states (illustrated by boxes in figure1) and seven possible transitions (illustrated by arrows in figure1). Given the Markov property, i.e. that the probability of future transitions only depends on the current state, the transition intensity of going to one state to the next is defined as:

$$h_i(t) = \lim_{\Delta t \to 0} \frac{P(Y(t + \Delta t) = a_i | Y(t) = b_i)}{\Delta t}$$

This is the instantaneous probability of the *i:*th transition of going from starting state a_i to finishing state b_i given that the process was in state a_i at time *t* i.e. this is the definition of a hazard rate. The multi-state model can therefore be specified as a combination of transition-specific survival models and the possible transitions defined by a transition matrix.

Illustration using simulated data

The simulated dataset “simulated_data.dta” contains 1000 observations with survival times and event indicators for the seven possible transitions stated by the multistate model. The simulation assumed a Weibull distribution for the different transitions between the states,

with shape and scale parameters selected by fitting Weibull models to the original data. The following STATA code require the latest version of user-written commands **`multistate`** and **`merlin`** that can be downloaded from the ssc archive. The code was written in Stata version 16.2 but is compatible with Stata versions 15.1 and higher.

```
// Install required packages by typing:
ssc install multistate
ssc install merlin

// Read simulated data (note that the working directory must first
// be set to where the data is stored locally).

use simulated_data.dta, clear

// Define transition matrix Ma (SFigure1)

mat tmat = (.,1,2,.,.,.,.,. ///
\.,.,.,3,4,.,.,. ///
\.,.,.,.,.,.,.,. ///
\.,.,.,.,.,5,6,. ///
\.,.,.,.,.,.,.,. ///
\.,.,.,.,.,.,.,7 ///
\.,.,.,.,.,.,.,. ///
\.,.,.,.,.,.,.,.)

// Name columns and row in the matrix

matrix colnames tmat = "CR" "R" "D1" "CR2" "D2" "R2" "D3" "D4"
matrix rownames tmat = "CR" "R" "D1" "CR2" "D2" "R2" "D3" "D4"
matrix list tmat

// Use msset to prepare the data

msset, id(id) transmat(tmat) states(first_relapse death_cr1 ///
second_cr death_relapse1 second_relapse death_cr2 death_relapse2) ///
times(time1 time2 time3 time4 time5 time6 time7)

// List event times for two patients

list id _from _to _start _stop _status _trans if inlist(id,4,10), noobs ///
sepby(id)

// Calculate how many patients underwent each possible transition and
// compare the numbers to the frequency matrix that msset returns

mat list r(freqmatrix)
count if _trans==1 & _status==1
count if _trans==2 & _status==1 //etc.


// Declare the data as survival data using stset. In the clock-forward
// Markov setting:

stset _stop, enter(_start) failure(_status=1)
```

```
// Now your data is stset, use msboxes to create a summary display of the
// transitions (Figure 1)
```

```
msboxes, transmat(tmat) id(id) xvalues(0.2 0.4 0.2 0.6 0.4 0.8 0.6 0.8) ///
yvalues(0.7 0.7 0.2 0.7 0.2 0.7 0.2 0.2) ///
statenames("CR" "R" "D1" "CR2" "D2" "R2" "D3" "D4") boxwidth(0.13)
```

In this paper, all transition-specific survival models were modeled using flexible parametric models, fitted on the log cumulative hazard scale, using restricted cubic splines to estimate the baseline hazard function. The number of degrees of freedom for the baseline hazard for each transition model was chosen by re-fitting the model with a range of knots (different number and localization) and comparing the AIC and BIC (Stable1). We illustrate this using the simulated dataset:

```
// For prediction: fit separate models for each transition and store them.
// Vary the degrees of freedom to find the optimum for each transition
// model, use the AIC and BIC to select the optimum number

forvalues i=1/7 {
      forvalues df = 1/5 {
            quietly stmerlin if _trans==`i', df(`df') distribution(rp)
            est store m`i'_`df'
      }
      qui count if _trans==`i'& _d==1
      est stats m`i'_*, n(`r(N)')
}
```

```
// Use predictms to calculate transition probabilities from fitted survival
models - for more details on the options see the help file for predictms

capture range temptime 0 11 100
predictms , transmat(tmat) models(m1_2 m2_1 m3_1 m4_2 m5_1 m6_1 m7_2) ///
timevar(temptime) prob visit ///
seed(32)
```

The Markov assumption can be relaxed by assuming a Markov renewal or clock-reset process, where time since entry in the current state is used as the underlying time scale for the process. Now the probability of future transitions not only depends on the current state but also on the time of entering the current state.

```
// reset time
gen double _newt = _stop - _start
```

```
// Declare the data as survival data using stset.

stset _newt, failure(_status=1)

// Refit the models

forvalues i=1/7 {
      forvalues df = 1/5 {
            quietly stmerlin if _trans==`i', df(`df') distribution(rp)
            est store m`i'_`df'
      }
      qui count if _trans==`i'& _d==1
      est stats m`i'_*, n(`r(N)')
}


// Use predictms to calculate transition probabilities from fitted survival
// models with option "reset" (see help predictms)

predictms , transmat(tmat) models(m1_1 m2_1 m3_1 m4_1 m5_2 m6_1 m7_1) ///
timevar(temptime) prob visit ///
reset /// //clock reset
seed(2830) ci m(20)

//Confidence intervals, I have changed to m(20) only for demonstration
//purpose as it can take a long time to run - default is m=200


// Rename variables created by msset to something more informative, e.g.,
// _visit* contains probabilities of ever visiting a state

// Probability of ever visiting state 2: First relapse (R1)
gen _visit_relapse = _visit_at1_1_2

// Probability of ever visiting state 3: Death in remission (D1)
gen _visit_cr_death = _visit_at1_1_3

// Probability of ever visiting state 4: Second remission (CR2)
gen _visit_cr2 = _visit_at1_1_4

// Probability of ever visiting state 5: Death after first relapse (D2)
gen _visit_relapse_death = _visit_at1_1_5

// Probability of ever visiting state 6: Second relapse (R2)
gen _visit_relapse2 = _visit_at1_1_6

// Probability of ever visiting state 7: Death in second remission (D3)
gen _visit_cr2_death = _visit_at1_1_7

// Probability of ever visiting state 8: Death after second relapse (D4)
gen _visit_relapse2_death = _visit_at1_1_8

// Generate Figure 2: Panels A, B, C, D
// A: From CR. What is the probability of remaining in CR (state 1) at
// different time points?
// What is the probability of having transitioned (from remission) to a
// first relapse or death at different time points?

//Stack probabilities
```

```
gen _visit_cr_death_stack = _visit_relapse + _visit_cr_death

twoway (area _visit_cr_death_stack temptime, sort ///
color(dkorange) lwidth(none)) ///
(area _visit_relapse temptime, sort ///
color(sandb) lwidth(none)) ///
(rarea _prob_at1_1_1_lci _prob_at1_1_1_uci temptime, sort ///
color(gs10) fi(50) lwidth(none)) ///
(line _prob_at1_1_1 temptime, sort ///
color(black) lpattern(solid)) ///
,ytitle("Probability") ///
xtitle("Years since attaining first remission") ///
subtitle("Probability of remaining in first remission") ///
legend(order(1 "Transitioned to death" ///
2 "Transitioned to first relapse") ///
col(1) size(small) symysize(*0.2) ///
region(lwidth(none) margin(medium) ///
color(ltbluishgray))) ///
ylabel(0(0.2)1.0,format(%3.1f) ///
angle(horizontal)) ///
xlabel(0(2)10) ///
name(figur2_A, replace)

//B: From First relapse. What is the probability of relapse (state 3) at
different time points?
//What is the probability of having transitioned (from having a relapse) to
a second remission or to //death at different time points?

//Stack probabilities
gen _visit_cr2_stack = _visit_relapse_death + _visit_cr2

twoway (area _visit_cr2_stack temptime, ///
sort color("141 159 13") lwidth(none)) ///
(area _visit_relapse_death temptime, sort ///
color("211 53 14") lwidth(none)) ///
(rarea _prob_at1_1_2_lci _prob_at1_1_2_uci temptime, sort ///
color(gs10) lwidth(none) fi(50)) ///
(line _prob_at1_1_2 temptime, sort ///
color(black) lpattern(solid)) ///
,ytitle("Probability")  ///
xtitle("Years since attaining first remission") ///
subtitle("Probability of first relapse") ///
legend(order(1 "Transitioned to second remission" ///
2 "Transitioned to death") ///
col(1) size(small) symysize(*0.2) ///
region(lwidth(none) margin(medium) ///
color(ltbluishgray))) ///
ylabel(0(0.05)0.2,format(%3.2f) angle(horizontal)) ///
xlabel(0 (2) 10) ///
name(figur2_B, replace)

//C: From Second remission. What is the probability of being in second
remission (state 4) at //different time points?
// What is the probability of having transitioned (from second remission)
to a second relapse or to //death at different time points?

//Stack probabilities

gen _visit_cr2_death_stack = _visit_cr2_death + _visit_relapse2
```

```
twoway (area _visit_cr2_death_stack temptime, sort ///
color(dkorange) lwidth(none)) ///
(area _visit_relapse2 temptime, sort ///
color(sandb) lwidth(none)) ///
(rarea _prob_at1_1_4_lci _prob_at1_1_4_uci temptime, sort ///
color(gs10) lwidth(none) fi(50)) ///
(line _prob_at1_1_4 temptime, sort ///
color(black) lpattern(solid)) ///
,ytitle("Probability")  ///
xtitle("Years since attaining first remission") ///
subtitle("Probability of second remission") ///
legend(order(1 "Transitioned to death" ///
2 "Transitioned to second relapse") ///
col(1) size(small) symysize(*0.2) ///
region(lwidth(none) margin(medium) ///
color(ltbluishgray))) ///
ylabel(0(0.02)0.1,format(%3.2f)angle(horizontal)) ///
xlabel(0 (2) 10) ///
name(figur2_C, replace)

//D: From second relapse: What is the probability of having a second
relapse at different time //points?
//What is the probability of having transitioned (from a second relapse) to
death?

twoway (area _visit_relapse2_death temptime, sort ///
color("211 53 14") lwidth(none)) ///
(rarea _prob_at1_1_6_lci _prob_at1_1_6_uci temptime, sort ///
color(gs10) lwidth(none) fi(50)) ///
(line _prob_at1_1_6 temptime, sort ///
color(black) lpattern(solid)) ///
,ytitle("Probability")  ///
xtitle("Years since attaining first remission") ///
subtitle("Probability of second relapse") ///
legend(order(1 "Transitioned to death") ///
size(small) symysize(*0.2) ///
region(lwidth(none) margin(medium) ///
color(ltbluishgray))) ///
ylabel(0(0.02)0.1,format(%3.2f) angle(horizontal)) ///
xlabel(0(2)10) ///
name(figur2_D, replace)

// Combine into one figure
graph combine figur2_A figur2_B figur2_C figur2_D, col(2)
graphregion(margin(zero)) name(figure2, replace)
```

Estimating the probability of remaining in remission by clinical subgroups

For estimating the probability of remaining in remission by clinical subgroups (Figure 3), we considered eight risk groups. The simulated data set contain a variable riskgroup with four categories.

```
//Create dummy variables: riskgroup1-riskgroup4

tab riskgroup, gen(riskgroup)
```

The transition rates were allowed to differ for each clinical subgroup through inclusion of interaction terms between group and the baseline hazard function (tvc() option).

```
//Fit separate models for each transition and store them

forvalues i = 1/7 {
stmerlin riskgroup2 riskgroup3 riskgroup4 if _trans==`i', df(3) ///
tvc(riskgroup2 riskgroup3 riskgroup4) dftvc(1) ///
distribution(rcs)
estimates store t`i'
}

//Use predictms to calculate transition probabilities from fitted survival
models - see help file for predictms for options

predictms, transmat(tmat) ///
models(t1 t2 t3 t4 t5 t6 t7) ///
at1() at2(riskgroup2 1) at3(riskgroup3 1) at4(riskgroup4 1) ///
timevar(temptime) prob ///
reset ///
seed(32)

capture drop prob*

// Stack probabilities


forvalues i = 1/4 {
capture drop prob*
gen prob_cr`i' = _prob_at`i'_1_1
gen prob_cr_2nd`i' = _prob_at`i'_1_1 + _prob_at`i'_1_4
gen prob_relapse`i'= _prob_at`i'_1_1 + _prob_at`i'_1_4 + _prob_at`i'_1_2
gen prob_relapse2nd`i'= _prob_at`i'_1_1 + _prob_at`i'_1_4 + ///
 _prob_at`i'_1_2 + _prob_at`i'_1_6
gen prob_relapse_death`i'= _prob_at`i'_1_1 + _prob_at`i'_1_4 + ///
_prob_at`i'_1_2 + _prob_at`i'_1_6 + _prob_at`i'_1_5 + _prob_at`i'_1_8
gen prob_cr_death`i'= _prob_at`i'_1_1 + _prob_at`i'_1_4 + ///
_prob_at`i'_1_2 + _prob_at`i'_1_6 + _prob_at`i'_1_5 + ///
_prob_at`i'_1_8 + _prob_at`i'_1_3  + _prob_at`i'_1_7


twoway (area prob_cr_death`i' temptime, sort color(dkorange)
lpattern(solid)) ///
(area prob_relapse_death`i' temptime, sort ///
color("211 53 14") lpattern(solid)) ///
(area prob_relapse2nd`i' temptime, sort color(maroon) lpattern(solid)) ///
(area prob_relapse`i' temptime, sort color(sandb) lpattern(solid)) ///
(area prob_cr_2nd`i' temptime, sort color("141 159 13") lpattern(solid))
///
(area prob_cr`i' temptime, sort color(emerald) lpattern(solid)) ///
,ytitle("Probability of states")  ///
xtitle("Years since attaining remission") ///
subtitle("Riskgroup `i'") ///
legend(order(6 "Remission after first line treatment" ///
4 "First relapse /// (alive)" ///
2  "Death after relapse (first + second)" ///
5 "Remission after ///first relapse" ///
3 "Second relapse (alive)" ///
1 "Death in remission ///(first + second)") ///
cols(3) symxsize(*0.5) colgap(*1.5) size(vsmall) ///
```

```
region(lwidth(none) margin(small) color(ltbluishgray))) ///
ylabel(0(0.25)1.0,format(%3.1f)) xlabel(0(2)10) ///
graphregion(margin(zero)) name(figure3_`i', replace)

}
//Combine subfigures into figure 3

grc1leg figure3_1 figure3_2 figure3_3 figure3_4, name(figure3, replace)
```

Difference in probability of remaining in remission and length of stay in remission

We also estimated the impact of risk factors (e.g. WHO performance status and elevated S-LDH) on the difference in the 2-year remission transition probabilities and in the length of stay in remission (Figure 4) adjusted for age, calendar year and sex.

We exemplify this by estimating the impact of riskgroup on the difference in the 2-year probability of remaining in remission and length of stay in remission adjusted by sex (female 0/1). The option "los" in predictms gives length of stay and the option "diff" gives differences.

```
//Fit model for transition 1 and 2 adjusting for female(0/1)

stmerlin riskgroup2 riskgroup3 riskgroup4 female if _trans==1, ///
distribution(weibull)
estimates store t1a

stmerlin riskgroup2 riskgroup3 riskgroup4 female if _trans==2, ///
distribution(weibull)
estimates store t2a

//Use predictms to calculate differences in transition probabilities and
length of stay from fitted survival models - see help predictms

predictms, transmat(tmat) ///
models(t1a t2a t3 t4 t5 t6 t7) ///
at1() at2(riskgroup2 1) at3(riskgroup3 1) at4(riskgroup4 1) ///
timevar(temptime) los diff prob /// //option diff gives differences
ci seed(32) m(20) //Confidence intervals, Note that m(20) is used only for
demonstration purpose as it can take a long time to run - default is m=200

// Rename the predicted differences in probabilities generated by predictms
// to something more informative

rename _diff_prob_at2_1_1 _diff_prob_CR_riskgrp2
rename _diff_prob_at2_1_1_lci _diff_prob_CR_riskgrp2_lci
rename _diff_prob_at2_1_1_uci _diff_prob_CR_riskgrp2_uci

rename _diff_prob_at3_1_1 _diff_prob_CR_riskgrp3
rename _diff_prob_at3_1_1_lci _diff_prob_CR_riskgrp3_lci
rename _diff_prob_at3_1_1_uci _diff_prob_CR_riskgrp3_uci

rename _diff_prob_at4_1_1 _diff_prob_CR_riskgrp4
rename _diff_prob_at4_1_1_lci _diff_prob_CR_riskgrp4_lci
rename _diff_prob_at4_1_1_uci _diff_prob_CR_riskgrp4_uci
```

```
// Rename the predicted "length of stay" generated by predictms to
something more informative

rename _diff_los_at2_1_1 _diff_los_CR_riskgrp2
rename _diff_los_at2_1_1_lci _diff_los_CR_riskgrp2_lci
rename _diff_los_at2_1_1_uci _diff_los_CR_riskgrp2_uci

rename _diff_los_at3_1_1 _diff_los_CR_riskgrp3
rename _diff_los_at3_1_1_lci _diff_los_CR_riskgrp3_lci
rename _diff_los_at3_1_1_uci _diff_los_CR_riskgrp3_uci

rename _diff_los_at4_1_1 _diff_los_CR_riskgrp4
rename _diff_los_at4_1_1_lci _diff_los_CR_riskgrp4_lci
rename _diff_los_at4_1_1_uci _diff_los_CR_riskgrp4_uci

// Figure 4

capture range xrange 0 3.4 100

gen x1 = 0.2
gen x2 = 1.2
gen x3 = 2.2
gen x4 = 3.2

gen ref_diff = 0

// Difference in probability of remission at t=2
twoway (line ref_diff xrange, sort color(black) lpattern(dash)) ///
(scatter ref_diff x1 if temptime==2, sort color(gs10)) ///
(scatter _diff_prob_CR_riskgrp2 x2 if temptime==2, sort color(gs0)) ///
(rspike _diff_prob_CR_riskgrp2_lci _diff_prob_CR_riskgrp2_uci x2 if ///
temptime==2, sort color(gs0) connect(l)) ///
(scatter _diff_prob_CR_riskgrp3 x3 if temptime==2, sort color(gs0)) ///
(rspike _diff_prob_CR_riskgrp3_lci _diff_prob_CR_riskgrp3_uci x3 ///
if temptime==2, sort color(gs0) connect(l)) ///
(scatter _diff_prob_CR_riskgrp4 x4 if temptime==2, sort color(gs0)) ///
(rspike _diff_prob_CR_riskgrp4_lci _diff_prob_CR_riskgrp4_uci x4 ///
if temptime==2, sort color(gs0) connect(l)) ///
, legend(off) yscale(range(-0.4 0.1)) ylabel(-0.4 (0.2) 0.0) ///
 xlabel(0.2 "Riskgroup 1 (ref)" 1.2 "Riskgroup 2" ///
 2.2 "Riskgroup 3" 3.2 "Riskgroup 4", angle(45) labsize(small)) ///
 ytitle("Difference* (95% CI)") ///
title("Difference in probability of remission after 2 years", ///
size(medsmall)) ///
graphregion(margin(zero sides)) name(fig4A_prob, replace)

 // Difference in length of stay in remission at t=2
twoway (line ref_diff xrange, sort color(black) lpattern(dash)) ///
(scatter ref_diff x1 if temptime==2, sort color(gs10)) ///
(scatter _diff_los_CR_riskgrp2 x2 if temptime==2, sort color(gs0)) ///
(rspike _diff_los_CR_riskgrp2_lci _diff_los_CR_riskgrp2_uci x2 if ///
temptime==2, sort color(gs0) connect(l)) ///
(scatter _diff_los_CR_riskgrp3 x3 if temptime==2, sort color(gs0)) ///
(rspike _diff_los_CR_riskgrp3_lci _diff_los_CR_riskgrp3_uci x3 if ///
temptime==2, sort color(gs0) connect(l)) ///
(scatter _diff_los_CR_riskgrp4 x4 if temptime==2, sort color(gs0)) ///
(rspike _diff_los_CR_riskgrp4_lci _diff_los_CR_riskgrp4_uci x4 if ///
temptime==2, sort color(gs0) connect(l)) ///
, legend(off) yscale(range(-0.4 0.1)) ///
ylabel(-0.4928 "-180" -0.32856 "-120" -0.2053 "-75" -0.082 "-30" ///
0 "0" 0.082 "30") ///
```

```
xlabel(0.2 "Riskgroup 1 (ref)" 1.2 "Riskgroup 2" ///
2.2 "Riskgroup 3" 3.2 "Riskgroup 4", angle(45) labsize(small)) ///
ytitle("Difference* (95% CI) (days)") ///
title("Difference in length of stay in remission after 2 years", ///
size(medsmall)) ///
graphregion(margin(zero sides)) name(fig4B_los, replace)

graph combine fig4A_prob fig4B_los, note("*adjusted for sex") ///
 name(figure4, replace)
```

SFigure1. Illustration of the multi-state model consisting of 7 transition models (arrows) and the transition matrix M_a.

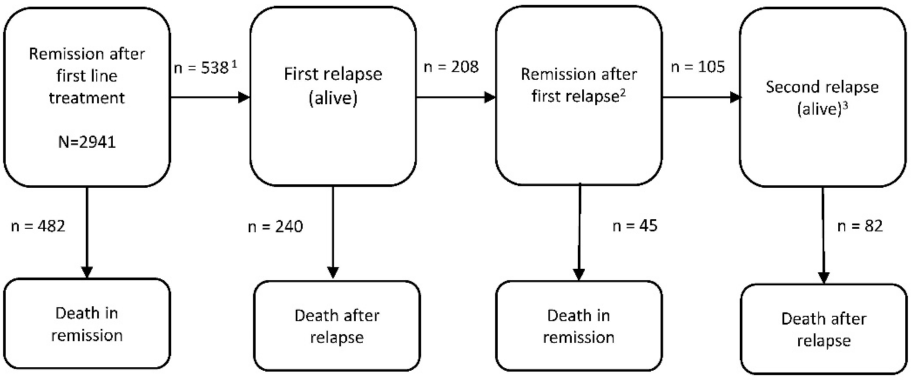

$$M_a = \begin{bmatrix} . & 1 & 2 & . & . & . & . & . \\ . & . & . & 3 & 4 & . & . & . \\ . & . & . & . & . & . & . & . \\ . & . & . & . & . & 5 & 6 & . \\ . & . & . & . & . & . & . & . \\ . & . & . & . & . & . & . & 7 \\ . & . & . & . & . & . & . & . \\ . & . & . & . & . & . & . & . \end{bmatrix}$$

STable 1: AIC and BIC for the seven transition models building up the multistate model with different number of knots and placements**.**

Transition	Degrees of freedom	Knots	Centile position of knots	AIC	BIC
1.	1 (=weibull)	0	No knots	4352.371	4364.344
(Remission after first	2	1	50	4195.912	4213.871
line treatment	**3**	**2**	**33, 67**	**4172.806**	**4196.752**
->	4	3	25, 50, 75	4174.98	4204.912
First relapse (alive))	5	4	20, 40, 60, 80	4175.764	4211.683
	6	5	17, 33, 50, 67, 83	4176.146	4218.052
2.	1 (=weibull)	0	No knots	4216.962	4228.935
(Remission after first	2	1	50	4197.263	4215.223
line treatment	3	2	33, 67	4196.338	4220.284
->	**4**	**3**	**25, 50, 75**	**4183.81**	**4213.743**
Death in remission)	5	4	20, 40, 60, 80	4184.601	4220.52
	6	5	17, 33, 50, 67, 83	4186.748	4228.654
3.	1 (=weibull)	0	No knots	535.3246	543.6343
(First relapse (alive)	2	1	50	382.212	394.6765
->	3	2	33, 67	378.9793	395.5988
Remission after first	**4**	**3**	**25, 50, 75**	**373.1436**	**393.9179**
relapse)	5	4	20, 40, 60, 80	373.5592	398.4883
	6	5	17, 33, 50, 67, 83	372.7267	401.8107
4.	1 (=weibull)	0	No knots	530.1018	538.4115
(First relapse (alive)	2	1	50	496.0954	508.56
->	**3**	**2**	**33, 67**	**495.6917**	**512.3111**
Death after relapse)	4	3	25, 50, 75	497.5644	518.3387
	5	4	20, 40, 60, 80	498.0563	522.9854
	6	5	17, 33, 50, 67, 83	499.3263	528.4103
5.	1 (=weibull)	0	No knots	461.0812	467.7563
(Remission after first	2	1	50	442.235	452.2476
relapse	**3**	**2**	**33, 67**	**440.1242**	**453.4744**
->	4	3	25, 50, 75	440.6068	457.2945
Second relapse	5	4	20, 40, 60, 80	442.5969	462.6221
(alive))	6	5	17, 33, 50, 67, 83	444.5345	467.8973
6.	1 (=weibull)	0	No knots	275.6744	282.3495
(Remission after first	**2**	**1**	**50**	**268.2731**	**278.2857**
relapse	3	2	33, 67	269.5912	282.9413
->	4	3	25, 50, 75	270.8949	287.5826
Death in remission)	5	4	20, 40, 60, 80	272.1312	292.1564
	6	5	17, 33, 50, 67, 83	273.4305	296.7933
7.	1 (=weibull)	0	No knots	206.7443	212.0522
(Second relapse	**2**	**1**	**50**	**195.5842**	**203.5461**
(alive)	3	2	33, 67	197.384	207.9998
->	4	3	25, 50, 75	196.6606	209.9304
Death after relapse)	5	4	20, 40, 60, 80	197.3271	213.2508
	6	5	17, 33, 50, 67, 83	198.2988	216.8765
